# Surgical management of ossifying fibroma in a 9-year-old Hungarian Vizsla: a case report and review of the literature

**DOI:** 10.3389/fvets.2024.1497077

**Published:** 2025-01-10

**Authors:** Romelia Pop, Alexandru-Flaviu Tăbăran, Iosif Vasiu, Joshua Milgram, Ciprian Andrei Ober

**Affiliations:** ^1^Department of Pathology, Faculty of Veterinary Medicine, University of Agricultural Sciences and Veterinary Medicine of Cluj-Napoca, Cluj-Napoca, Romania; ^2^Department of Anesthesiology and Surgery, University of Agricultural Sciences and Veterinary Medicine, Cluj-Napoca, Romania; ^3^Department of Small Animal Surgery, The Koret School of Veterinary Medicine, The Hebrew University of Jerusalem, Rehovot, Israel

**Keywords:** fibroma, ossifying, zygomatic, radiology, histopathology, dog

## Abstract

Ossifying fibroma (OF) is a rare, benign fibro-osseous neoplasm that primarily originates from membranous bones. While most frequently documented in equines, OF has also been reported in other species, including dogs, though it remains uncommon. The condition poses significant diagnostic challenges due to its ambiguous presentation, often requiring differentiation from other benign and malignant intraosseous lesions. This case report describes an ossifying fibroma localized to the zygomatic bone in a 9-year-old Hungarian Vizsla. A zygomatic arch ostectomy was successfully performed, and long-term follow-up was excellent. This is only the second documented case of zygomatic localization of OF in a dog, highlighting the rarity of this presentation. The discussion emphasizes the importance of distinguishing OF from other proliferative fibro-osseous lesions, such as fibrous dysplasia (FD) and cemento-osseous dysplasia (COD), and considering the potential for malignancies, such as low-grade osteosarcoma (LG-OSA), to mimic these benign growths. This case contributes valuable insights to the limited veterinary literature on ossifying fibroma, particularly regarding its atypical presentations in canine patients.

## 1 Introduction

**Figure 1:**
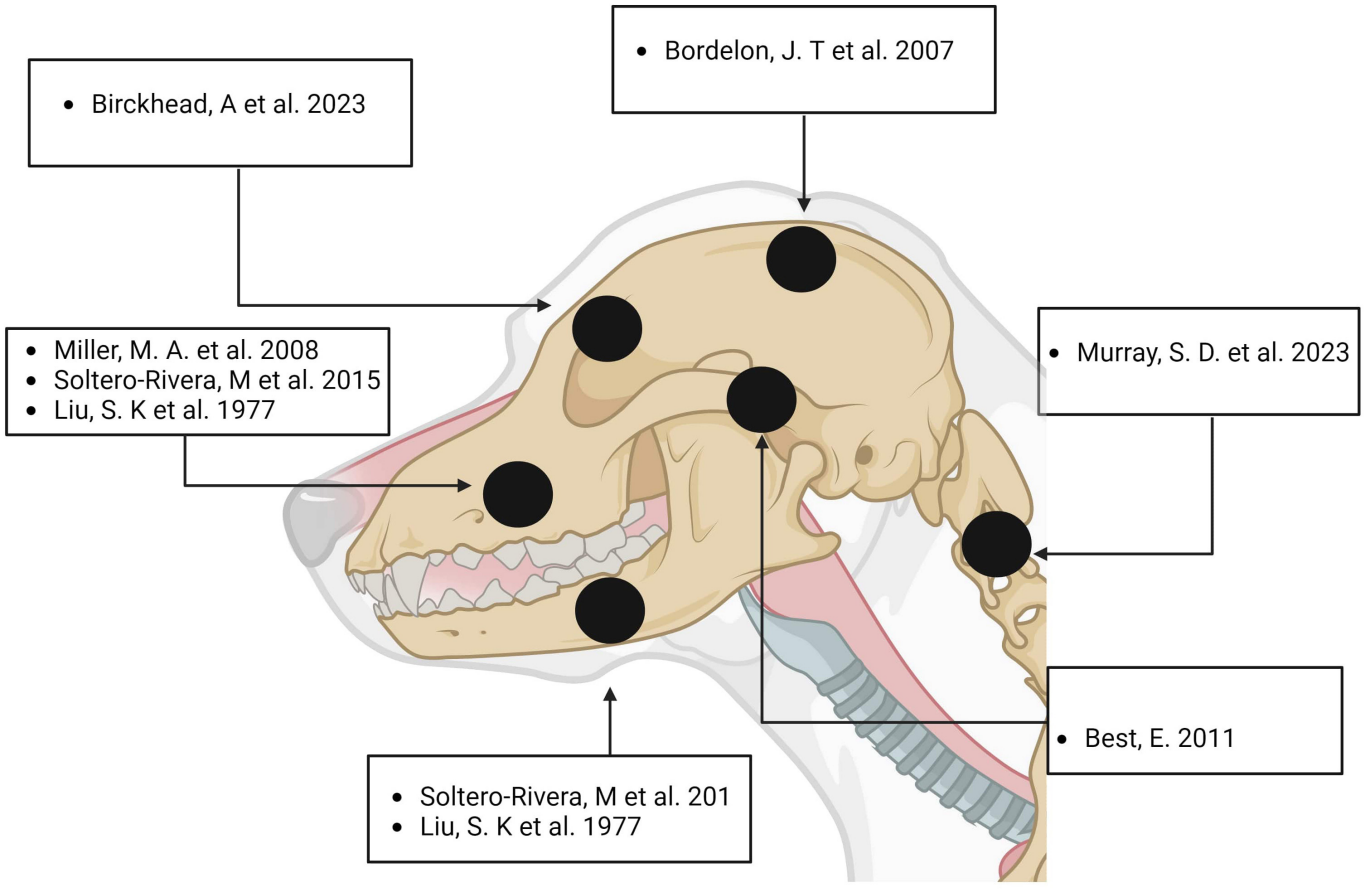
Distribution of Ossifying Fibroma in the Cranio-Cervical Region of Dogs. Created with Biorender.

Ossifying fibroma (OF) represents a rare and benign fibro-osseous neoplasm (1) that primarily arises from membranous bones (2). Its infrequent occurrence and ambiguous clinical presentation pose significant diagnostic and therapeutic challenges in veterinary medicine. OF is most commonly found in equines, particularly horses (3–7), but it can also be observed in other species such as dogs (1, 8, 9) and cats (10). Rare instances have been reported in rabbits (11), llamas (12), and canaries (13) (see Table 1). In humans, according to the World Health Organization (WHO), the most frequent localization of ossifying fibroma is in the posterior mandible (14). Similarly, literature on canine cases indicates that the most common sites of ossifying fibroma are the mandible and maxilla (see Figure 1). This case report details the occurrence of ossifying fibroma in a 9-year-old Hungarian Vizsla with zygomatic localization. Through this detailed case study, we aim to contribute to the limited veterinary literature on ossifying fibroma, providing valuable insights for clinicians encountering similar cases in their practice. To the authors' knowledge, this is the second report of an ossifying fibroma with zygomatic bone localization in this species, the first one being described by Best, E. in a Corgi (15).

**Table 1:** Documented cases of ossifying fibroma in dogs: breed, age, and localization.

**No**.	**Breed**	**Age (years)**	**Localization**	**References**
1.	Pembroke Welsh corgi	1.8	Zygomatic arch	(15)
2.	Mixed breed	15	Left hemimandible	(9)
3.	Pembroke Welsh corgi	3	C6 cervical vertebra	(8)
4.	Australian Terrier	6	Left calvarium	(18)
5.	NS	7.5	Left caudal maxilla	(1)
6.	NS	13	Left rostral maxilla	(1)
7.	NS	12	Left caudal mandible	(1)
8.	Kelpie cross	10	Frontal sinus	(19)
9.	NS	NS	Maxilla (no specific location mentioned)	(20)
10.	NS	NS	Maxilla (no specific location mentioned)	(20)
11.	NS	NS	Mandible (no specific location mentioned)	(20)
12.	NS	NS	Mandible (no specific location mentioned)	(20)
13.	Golden retriever	9	Right mandible	(21)

NS, not specified.

## 2 Case description

### 2.1 Clinical examination

**Figure 2:**
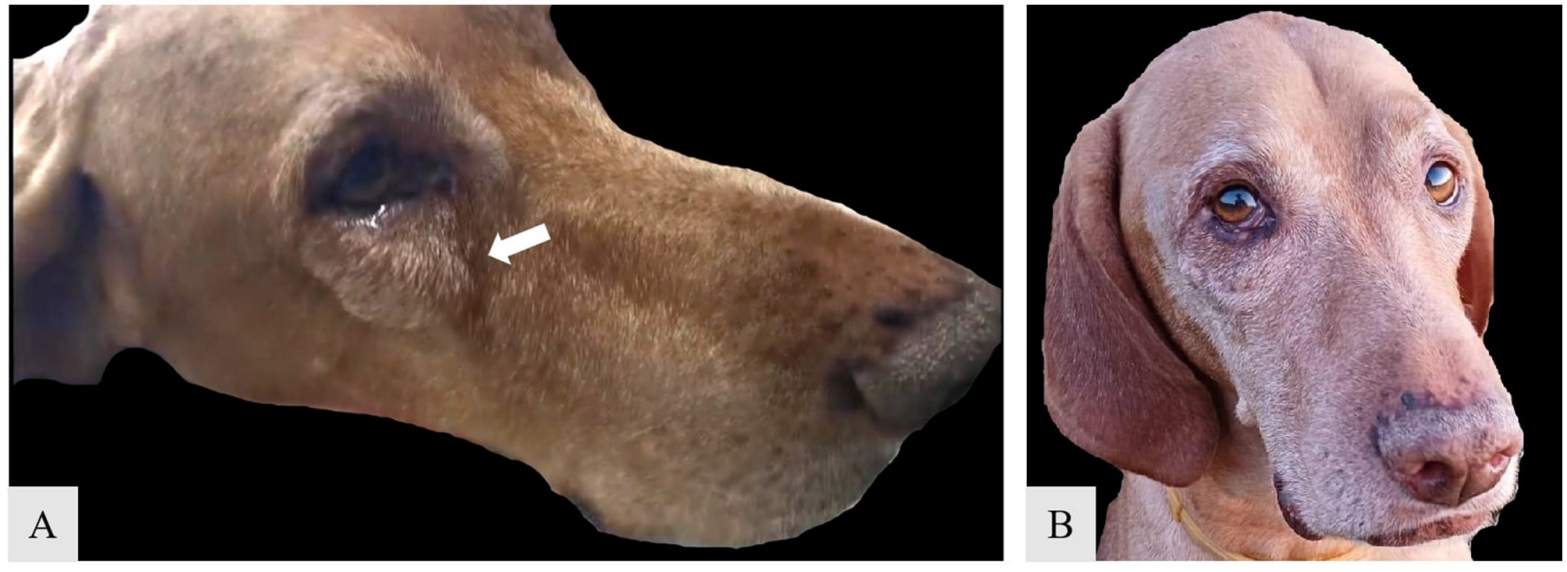
Preoperative appearance of neoplastic growth on the zygomatic arch in male Hungarian Viszla [**(A)** white arrow]; Ten-months postoperative aspect of the zygomatic area **(B)**.

**Figure 3:**
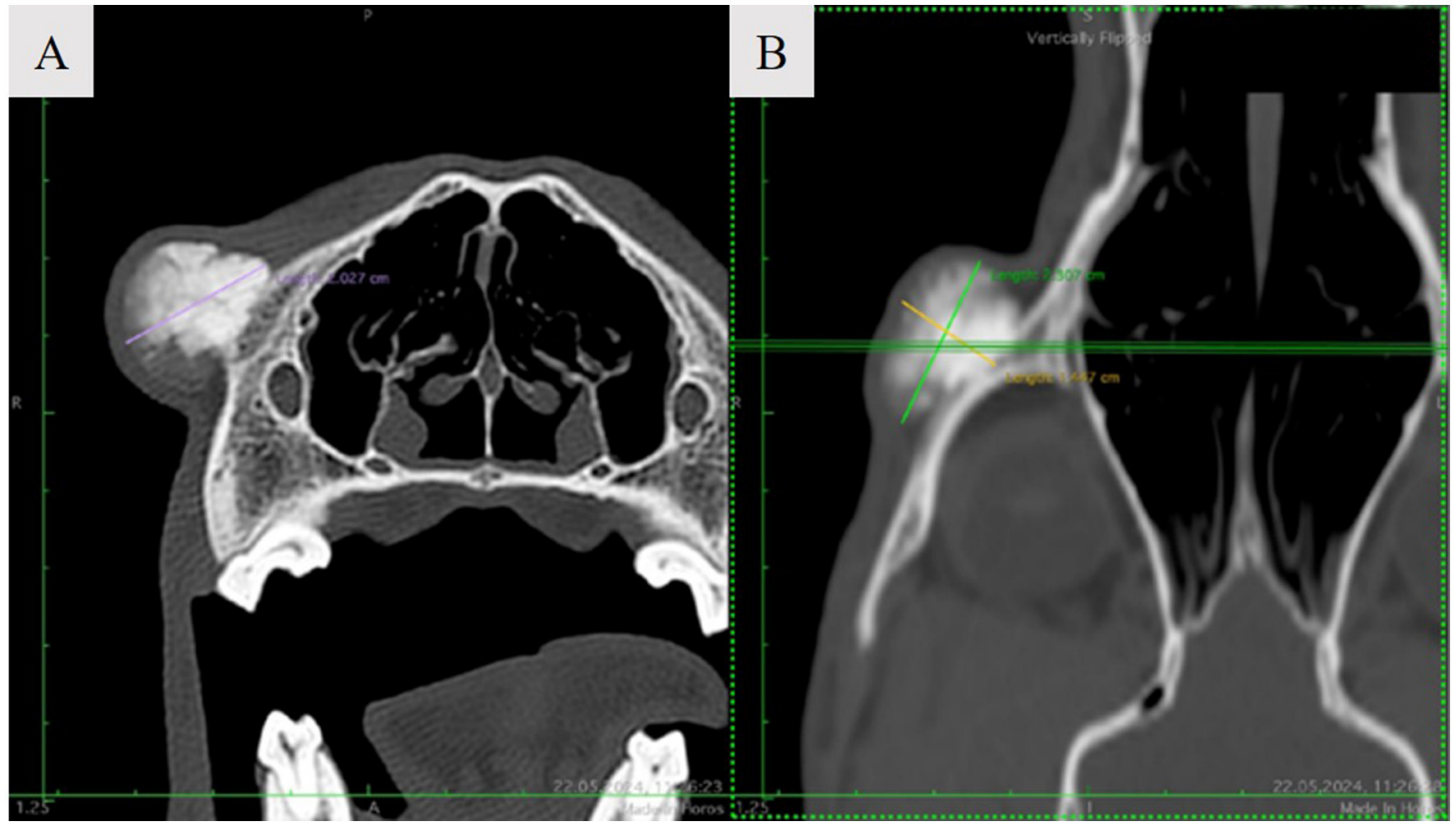
Noncontrast axial **(A)** and dorsal **(B)** CT image demonstrating a focal, round, expansile bone cortical lesion of the right zygomatic arch.

A 9.4-year-old male Hungarian Vizsla was referred to the Department of Surgery and Intensive Care for evaluation of a slow-growing zygomatic arch mass near the right eye, present for 2 years (see Figure 2A). Upon examination, the mass measured ~2.5 cm in diameter, with a multilobulated, firm, non-mobile, and non-painful consistency upon palpation. Occasional signs included blepharospasm, photosensitivity, hyperemic conjunctiva, and purulent epiphora. Non-contrast computed tomography (CT) revealed a focal, round, expansile bone lesion on the right zygomatic arch, measuring 2.0 × 2.3 × 1.5 cm (L, H, W) (see Figure 3). The inner bone structure, nasolacrimal duct, and teeth were unaffected, and no lymphadenopathy was noted. Thoracic CT showed no evidence of metastasis. Given the lesion's slow growth, the owner opted for an excisional biopsy. Cefazolin (22 mg/kg IV) was administered preoperatively. The surgical approach was well documented (16), and the cosmetic outcome was excellent following the zygomatic arch ostectomy. The procedure involved an incision of the skin and temporalis muscle aponeurosis along the dorsal margin of the zygomatic arch. Both cranial and caudal osteotomies were performed with an oscillating saw, preserving the orbital ligament. Histopathological analysis of the excised tissue confirmed the diagnosis of ossifying fibroma. The dog's recovery from surgery and anesthesia was uneventful. Postoperative pain management was maintained using a constant rate infusion (CRI) of lidocaine (20 μg/kg/h) combined with ketamine (10 μg/kg/h) and metamizole (25 mg/kg IV every 8 h). The dog was discharged with robenacoxib (2 mg/kg SC every 24 h). The owner was advised to provide a soft kibble diet, avoid toys or mouth play, and restrict the dog's exercise to short-lead walks for two weeks.

### 2.2 Histopathological examination

**Figure 4:**
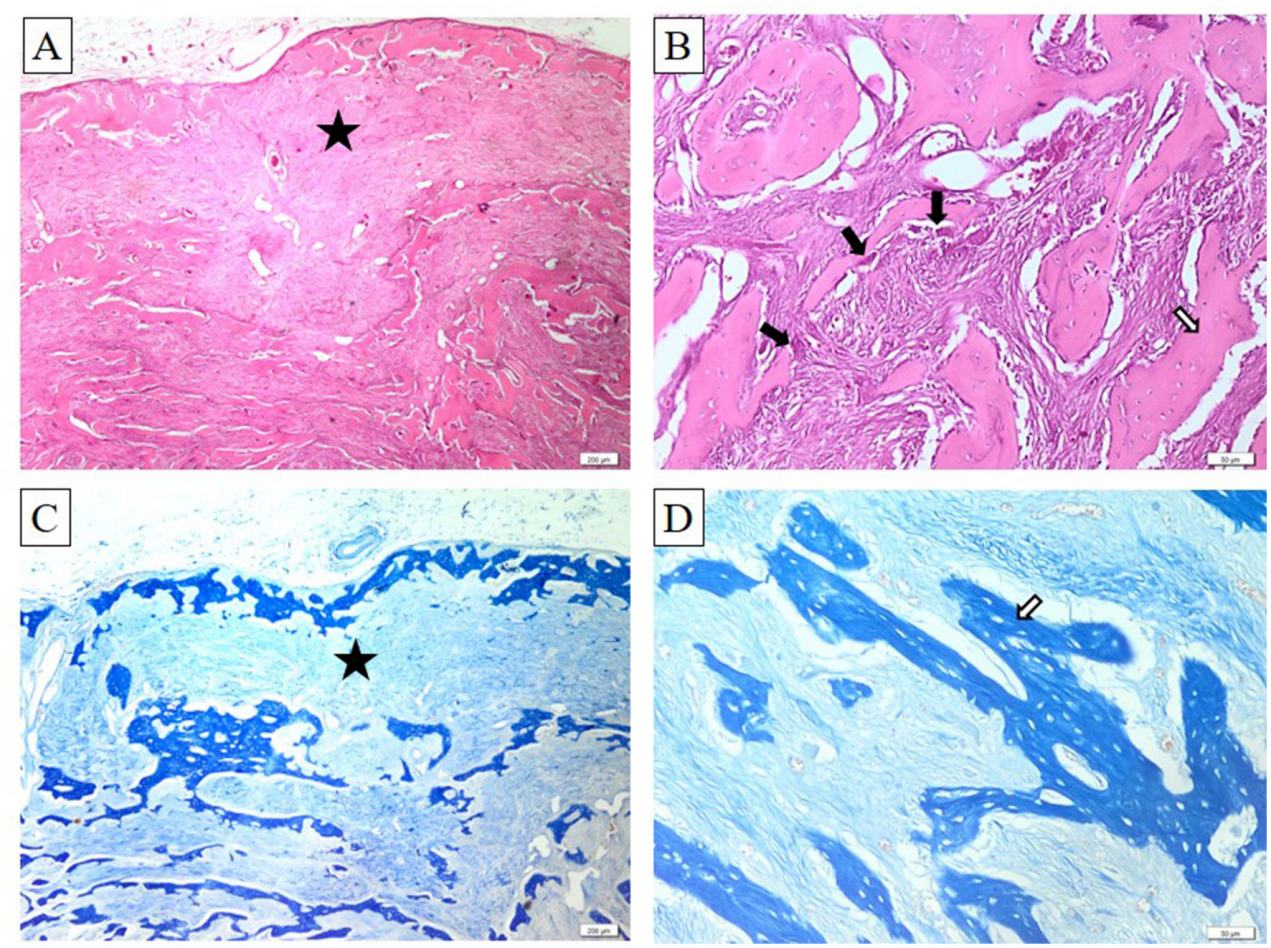
Ossifying fibroma. Bony trabeculae (white arrows) bordered by a single layer of osteoblasts (black arrows) and embedded within abundant fibrous connective tissue (black asterisk). Within this connective tissue, numerous well-differentiated spindle-shaped fibroblasts are dispersed. H&E stain **(A, B)**, Masson's trichrome stain **(C, D)**. Ob. ×4 **(A, C)** and ob. ×20 **(B, D)**. Barr 50 μm **(B, D)** and 200 μm **(A, C)**.

For histological analysis, the mass was fixed in 10% neutral buffered formalin (NBF) for 24 h, followed by decalcification in a rapid decalcifier for 5 days. The tissue was then routinely processed for paraffin embedding. Sections, 2 micrometers thick, were cut and stained with hematoxylin and eosin (H&E). Histopathological examination revealed a well-demarcated mass composed of a fibrous component consisting of spindle-shaped fibroblasts arranged in a whorled or storiform pattern, embedded within a collagenous stroma. Interspersed throughout the fibrous stroma were varying amounts of mineralized material, including woven bone, lamellar bone, and cementum-like calcifications. The mineralized component often appeared as trabeculae of osteoid and mature bone, occasionally rimmed by osteoblasts. These trabeculae were typically surrounded by osteoclast-like giant cells involved in bone remodeling. The transition between the fibrous tissue and the mineralized material was gradual, with no signs of anaplasia or atypia. The presence of well-formed bone trabeculae within a cellular fibroblastic stroma is characteristic of ossifying fibroma. Additionally, areas of hemorrhage and cystic degeneration were observed in some cases (see Figure 4).

### 2.3 Follow-up

Two weeks after discharge, the incision site showed proper healing, with a good cosmetic outcome. The owner reported normal prehension and behavior. The dog was bright, alert, tolerated food and water well, and was walked daily on a leash. Monthly follow-ups were conducted via telephone, and the owner was instructed to return for in-person evaluations at three-month intervals during the first year post-surgery. At 10 months after the procedure, a clinical examination revealed no signs of tumor recurrence (Figure 2B), and the owner reported no changes in the dog's eating, drinking, or behavior. Twelve months after surgery, the owner was very satisfied with the cosmetic appearance and the comfort of the right eye.

## 3 Discussion

Ossifying fibroma (OF) is a rare, benign fibro-osseous neoplasm that requires careful differentiation from other benign intraosseous proliferative fibro-osseous lesions (PFOLs). PFOLs are characterized by the replacement of normal bone with a fibrous matrix containing varying degrees of mineralization and ossification (1). In humans, this category includes conditions such as ossifying fibroma (OF), fibrous dysplasia (FD), and cemento-osseous dysplasia (COD) (17). It is important to recognize that some malignant lesions, like low-grade osteosarcoma (LG-OSA), can mimic these benign growths, especially in the skull.

A review of the literature highlights the rarity of ossifying fibroma across species, with most cases documented in equines, particularly horses (3–7). In small animals, reports are limited, although ossifying fibroma has been observed in dogs (1, 8, 9, 18–21) and cats (10). This limited occurrence poses challenges in diagnosis and treatment, particularly when the lesion arises in atypical locations, such as the zygomatic bone, as in the case presented here. The uniqueness of this case—only the second report of such localization in a dog—emphasizes the need for continued documentation and study of these rare cases to enhance understanding and management of OF in veterinary practice.

In veterinary medicine, other potential differential diagnoses for intraosseous lesions, besides true PFOLs like OF and FD, include osteoma, osteitis/osteomyelitis, fibrous osteodystrophy, conventional osteosarcoma (OSA), and multilobular tumor of bone (MLTB). FD is a rare benign condition in which fibrous tissue replaces normal bone, leading to deformities and swelling. It commonly affects young animals, with expansile growth potentially causing decreased bone strength and pathologic fractures, often in the skull and jawbones. Radiographically, fibrous dysplasia typically presents a more homogeneous “ground glass” appearance, lacking the well-defined margins characteristic of ossifying fibroma (17). Cemento-osseous dysplasia (COD) primarily affects the jawbones (maxilla and mandible) and is characterized by a disorganized mixture of fibrous tissue, irregular bone, and cementum-like material (22). However, in our case, the lesion was uniquely located in the zygomatic bone, underscoring the importance of careful diagnostic evaluation to distinguish ossifying fibroma from other intraosseous lesions. Given the rarity of OF in dogs and its variable presentation, this case report contributes valuable insights to the limited veterinary literature. The zygomatic localization presents a unique diagnostic challenge, requiring a comprehensive understanding of potential differential diagnoses. Since the literature primarily documents ossifying fibroma in the mandible and maxilla of dogs (1), this case highlights the importance of considering less common sites when diagnosing PFOLs in small animals. Continued reporting and review of such cases are critical to refining diagnostic and therapeutic approaches for ossifying fibroma and similar lesions in veterinary practice.

## Data Availability

The original contributions presented in the study are included in the article/supplementary material, further inquiries can be directed to the corresponding author.
